# Dynamics Analysis of Firing Patterns in Pre-Bötzinger Complex Neurons Model

**DOI:** 10.3389/fncom.2021.591037

**Published:** 2021-06-15

**Authors:** Quan Yuan, Jieqiong Xu, Huiying Chen

**Affiliations:** School of Mathematics and Information Science, Guangxi University, Guangxi, China

**Keywords:** pre-Bötzinger complex, bursting, bifurcation analysis, fast and slow analysis, simplified model

## Abstract

Pre-Bötzinger complex (PBC) neurons located in mammalian brain are the necessary conditions to produce respiratory rhythm, which has been widely verified experimentally and numerically. At present, one of the two different types of bursting mechanisms found in PBC mainly depends on the calcium-activated of non-specific cation current (*I*_*CaN*_). In order to study the influence of *I*_*CaN*_ and stimulus current *I*_*exc*_ in PBC inspiratory neurons, a single compartment model was simplified, and firing patterns of the model was discussed by using stability theory, bifurcation analysis, fast, and slow decomposition technology combined with numerical simulation. Under the stimulation of different somatic applied currents, the firing behavior of neurons are studied and exhibit multiple mix bursting patterns, which is helpful to further understand the mechanism of respiratory rhythms of PBC neurons.

## Introduction

In mammals, breathing is a continuous rhythmic behavior, which can be carried out autonomously and rhythmically. This rhythmic activity provides self-regulation for gases in blood and tissues, and combines breathing with other movement behaviors to make blood in animals reach a steady state (Khoo, 2000). Firing is very common in the nervous system, which is one of the main ways to encode neural information and usually has important functional meanings. The firing patterns of neurons are very rich and can be divided into two categories: bursting and spiking. Izhikevich (2000) discussed the excitability, spiking and bursting of neurons by using the bifurcation theory, and expounded the dynamic characteristics of bursting combined with simulation. At the same time, a new classification basis of bursting was presented, and the bursting discovered in this article was also classified based on this criterion. Among them, bursting is the main way of information transmission, so studying the firing patterns generated by neurons is of great guiding significance to the reception, transmission, and processing of neural information (Johnson et al., 2001).

At present, a large number of experiments have shown that pre-Bötzinger complex (PBC) is a necessary factor for the generation of respiratory rhythms, and there are excitatory respiratory neurons in PBC, which have bursting characteristics (Butera et al., 1999; Zylbertal et al., 2017; Lv et al., 2019). Rubin (2006) studied the network model of synaptic connections through excitable intermediate neurons and explained firing phenomenon of PBC. Bertram and Rubin (2017) and Rubin et al. (2018) studied the parameter range that affects bursting in PBC neural network by using fast-slow analysis and bifurcation theory, classified different bursting patterns in parameter space, and explained various bursting in detail by combining physical and biological systems. Kiehn (2016) turned to study the effect of ion change on neuron firing by observing the activity of single PBC neuron under the change of potassium and calcium ions, which was proved that the bursting of mammalian medulla cells was the key to the generation of respiratory rhythms combined with clinical experiments. Besides, the firing frequency of PBC was controlled by changing the concentration of extracellular potassium ions, and the conclusion was consistent with the model prediction in Neusch et al. (2006).

Different neuron models can also produce a variety of firing phenomena, and PBC is one of them. Toporikova and Butera (2011) identified two types of firing patterns in the model by coupling inspiratory PBC neurons, and found that the bursting mechanisms depend on the changes of persistent sodium ions (*NaP*) and calcium ions (*Ca^2+^*), respectively, which established a single compartment neuron model with two bursting mechanisms (also called TB model). Park and Rubin (2013) discovered the mixed bursting phenomenon in PBC by controlling calcium activated non-specific cationic (*CAN*) based on the TB model. Duan et al. (2012) and Wang et al. (2018) then investigated the mixed bursting in a single compartment PBC, and the results showed that the TB model could show various types of mixed bursting in the same period. In addition, the influence of potassium conductance *g*_*K*_ and leakage conductance *g*_*L*_ on the bursting was also described.

In this study, dendritic subsystem in TB model is deleted, and only the somatic subsystem is retained (Femando et al., 2004; Feldman and Del Negro, 2006; Toporikova and Butera, 2011) are available for the introduction of dendritic sub-model and somatic sub-model). There are two main reasons for deleting dendritic subsystem. Firstly, research on dendritic subsystem has been involved in a lot of literature (Femando et al., 2004; Frank et al., 2018; Lai et al., 2018). Moreover, there are too many parameters of dendritic subsystem in the model, which is not convenient for specific experiments. Secondly, dendritic subsystem affects the somatic subsystem through calcium concentration, while the somatic subsystem does not affect the calcium subsystem (Lv et al., 2019), and the range of calcium concentration in dendritic subsystem is very small, which can be considered as a constant relative to the somatic subsystem. The firing behavior of PBC neurons is discussed by applying external stimulus current to somatic subsystem (Lv and Ma, 2016). Then, the parameter space is divided into different regions by using the two-parameter bifurcation analysis, and the bursting characteristics of the firing region are emphatically discussed. Finally, the slow variables were parameterized by fast and slow dynamics analysis, and three types of blasting were found by taking different parameters of the fast subsystem. The structure of this article is as follows: Chapter 2 will introduce the mathematical model. The effect of calcium activated non-specific cationic conductance *g*_*cantot*_ on the firing of PBC neurons based on non-stimulated and stimulus currents were discussed in chapters 3 and 4, respectively. In chapter 5 fast and slow analysis is applied to find the types of bursting in neurons, and making a relevant conclusion at the end of the article.

## Model

**FIGURE 1:**
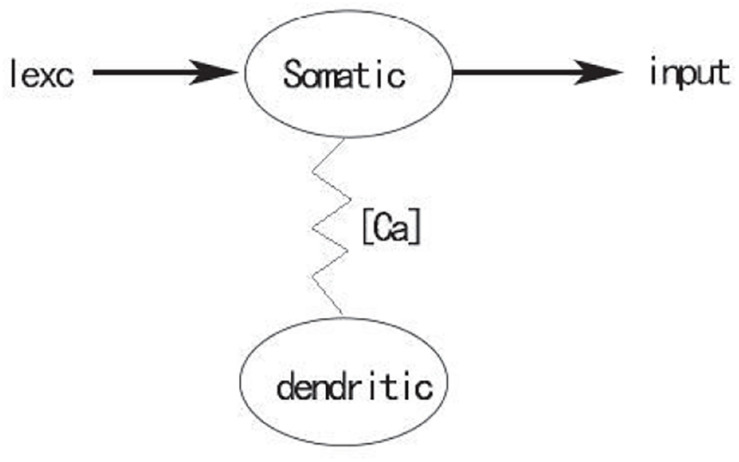
Schematic diagram of somatic-dendritic model.

In this study, a single compartment model of PBC inspiratory neurons based on calcium activated non-specific cationic conductance was used. This model is a simplification of the two-compartment model (also known as TB model) established by Toporikova and Butera (2011), in which dendritic subsystem is deleted, and only the somatic subsystem is studied. As shown in Figure 1, the somatic subsystem and the dendritic subsystem are connected through [*Ca*]. The former contains coupling variables [*Ca*], while the latter does. not include variables of the somatic subsystem, indicating that the somatic subsystem will be influenced by the dendritic subsystem through [*Ca*], while the dendritic subsystem is completely independent of the somatic subsystem. By applying stimulation current to the somatic compartment, the influence of external input on PBC neuron output can be observed, which shows the transition of different firing patterns. Moreover, stimulation current *I*_*exc*_ is considered in the model, which is finally composed of three differential equations:

(1) $$\dot{v}=\left(-I_{N\,a}-I_{K}-I_{N\,a\,P}-I_{L}-I_{C\,a\,N}-I_{e\,x\,c}\right)/C$$

(2) $$\dot{n}=\left(n_{\infty }\,\left(v\right)-n\right)/\tau_{n}\,\left(v\right)$$

(3) $$\dot{h}=\left(h_{\infty }\,\left(v\right)-h\right)/\tau_{h}\,\left(v\right)$$

where, *v* is the membrane voltage, *n* and *h* are the state variables of potassium and calcium ion gate, respectively, *I*_*Na*_, *I*_*K*_, *I*_*L*_, *I*_*NaP*_ and *I*_*CaN*_ are, respectively, sodium, potassium, leakage, persistent sodium and calcium activated non-specific cation current, and the expression is:

$$I_{N\,a}=g_{N\,a}\cdot m_{\infty }^{3}\cdot \left(1-n\right)\cdot \left(v-v_{N\,a}\right),$$

$$I_{N\,a\,P}=g_{N\,a\,P}\cdot m\,p_{\infty }\cdot \left(v-v_{N\,a}\right),$$

$$I_{K}=g_{K}\cdot n^{4}\cdot \left(v-v_{K}\right),$$

$$I_{L}=g_{L}\cdot \left(v-v_{L}\right),$$

$$I_{C\,a\,N}=g_{c\,a\,n\,t\,o\,t}\cdot \left(v-v_{N\,a}\right)$$

Steady state and time functions of voltage dependent activation and deactivation are:

$$\begin{array}{c} m_{\infty }\,\left(v\right)=\frac{1}{1+\exp\left(\frac{v-\theta_{m}}{\sigma_{m}}\right)}\\ m\,p_{\infty }\,\left(v\right)=\frac{1}{1+\exp\left(\frac{v-\theta_{m\,p}}{\sigma_{m\,p}}\right)} \end{array},\begin{array}{c} n_{\infty }=\frac{1}{1+\exp\left(\frac{v-\theta_{n}}{\sigma_{n}}\right)}\\ h_{\infty }=\frac{1}{1+\exp\left(\frac{v-\theta_{h}}{\sigma_{h}}\right)} \end{array},\begin{array}{c} \tau_{n}\,\left(v\right)=\frac{{\bar{\tau }}_{n}}{\cosh\left(\frac{v-\theta_{n}}{2\,\sigma_{n}}\right)}\\ \tau_{h}\,\left(v\right)=\frac{{\bar{\tau }}_{h}}{\cosh\left(\frac{v-\theta_{h}}{2\,\sigma_{h}}\right)} \end{array}$$

A detailed description of the parameters is given in Park and Rubin (2013) and Wang and Rubin (2016). All parameters are given in Table 1. The numerical software used in this article is mainly XPPAUT and MATLAB, and the fourthorder Runge-Kutta algorithm is used with a step size of 0.1.

**TABLE 1:** The default parameter.

Parameter	Value	Parameter	Value	Parameter	Value	Parameter	Value
*C*	21 μF	*g*_*cantot*_	0.02 nS	θ*_*m*_*	−34mV	σ*_*n*_*	−4 mV
*g*_*na*_	1.8 nS	*v*_*na*_	50 mV	σ*_*m*_*	−5mV	θ*_*h*_*	−48 mV
*g*_*k*_	4.2 nS	*v*_*k*_	−85 mV	θ*_*mp*_*	−40mV	σ*_*h*_*	5 mV
*g*_*l*_	2.3 nS	*v*_*l*_	−58 mV	σ*_*mp*_*	−6mV	τ_*h*_	10000 ms
*g*_*nap*_	3 nS	τ_*n*_	10 ms	θ*_*n*_*	−29mV		

## Firing Analysis Based on Calcium Activated Conductance Gcantot Without Stimulus Current (*I_exc_* = 0)

The form of non-specific calcium activation current in TB model is *I_*CaN*_* = *g*_*CaN*_⋅*f*([Ca])⋅(*v*-*v*_*na*_), where *g*_*CaN*_ is the non-specific calcium activation conductance and *f* is the calcium activation function related to calcium concentration. In this article, deleting the dendrite subsystem of the calcium-containing subsystem is equivalent to taking the calcium concentration constant as a constant, so *g_*cantot*_* = *g*_*CaN*_⋅*f*([Ca]). In addition, there has been a study on bursting caused by external stimulation current in Franaszczuk et al. (2003). Brain stimulation is used to regulate or terminate epileptic seizures, and two models of recurrent bursting are established. It is found that the ability of this external stimulation to terminate recurrent bursting may depend on identifiable parameters in the model, and it is known that the duration of neuronal bursting has a great correlation with the intensity of stimulation current input, which is almost consistent with the results in Lv and Ma (2016).

When the external stimulation current *I_*exc*_* = 0, that is, there is no external stimulation current. Pace et al. (2007) studied the firing pattern of glutamate receptor in newborn mice based on the non-specific calcium activated cation current *I*_*CaN*_. It has been generally believed that *I*_*CaN*_ has an important influence on the firing patterns, and the conductance *g*_*cantot*_ is an important parameter that determines the current and plays a vital role in the influence of membrane potential, thus *g*_*cantot*_ is taken as a parameter to study the firing pattern of respiratory rhythms of neurons in PBC.

**FIGURE 2:**
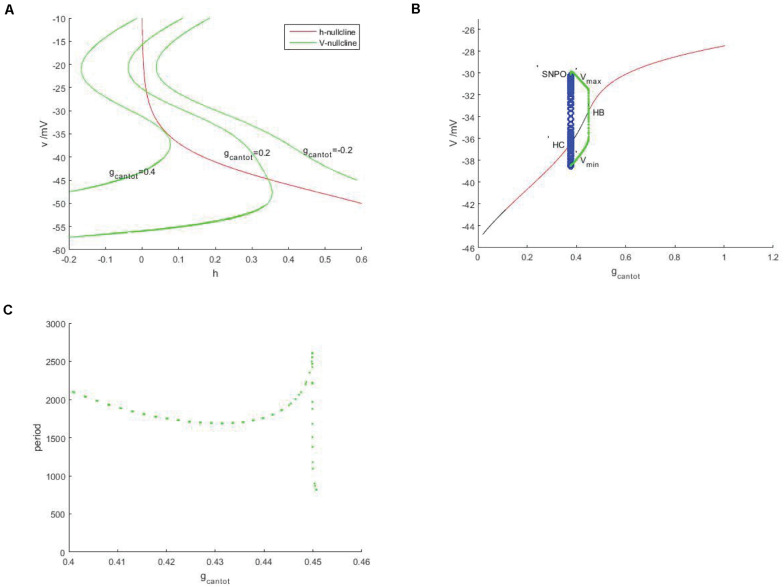
Neural model based on unstimulated current (*I_*exc*_* = 0). **(A)** Nullcine of the system in the (*h*,*v*) plane, red is *h*-nullcline, green is *v*-nullcline with different *g*_*cantot*_ values, from right to left, *g_*cantot*_* = –0.2, *g_*cantot*_* = 0.02, and *g_*cantot*_* = 0.4. **(B)** Bifurcation diagram of the parameter *g*_*cantot*_. The black and red represent the equilibrium point of instability and stability, respectively, the blue hollow circle represents the unstable periodic orbit, and the green solid circle represents the stable closed orbit. **(C)** Changes of respiratory rhythms cycle of neurons in PBC with *g*_*cantot*_.

In Figure 2A, red is the *h*-nullcline in the plane (*h*,*v*), which is monotonically decreasing and is not affected by *g*_*cantot*_, while green is the *v*-nulcline, which is S-shaped. With the increase of *g*_*cantot*_, the left and right knee values move to the left and decrease in amplitude. In addition, the number of intersections between the branches of the *v*-nullcline and the *h*-nullcline (the equilibrium point of the system) went from 0 to 2 and back to 1. It can be assumed that the number of intersections will still be 0 if *g*_*cantot*_ continues to increase. Therefore, there must be a bifurcation with respect to the parameter *g*_*cantot*_. As shown in Figure 2B, black represents the unstable equilibrium points, red represents the stable equilibrium points, green and blue represent the stable and unstable periodic orbits, respectively. According to the stability of the equilibrium, the bifurcation curve has four regions, respectively.

I (0,0.1034) II (1.1034,0.3775) III (0.3775,0.4507) IV (0.4507,1.006).

Instability and stability appear alternately, the intersection points of these regions are Hopf bifurcations, embarking from one of them (take *g_*cantot*_* = 0.4507 in Figure 2B), a stable limit cycle appears, then becomes unstable at *g_*cantot*_* = 0.3775, and finally returns to the equilibrium curve. These two points are, respectively, saddle-node bifurcation of periodic orbits (SNPO) and homoclinic bifurcation (HC) on the invariant cycle. It should be noted that there is also a saddle node bifurcation below the periodic orbit bifurcation curve. From the selected Hopf bifurcation point, the equilibrium changes from unstable to stable and produces a stable limit cycle, which shows that it is also a supercritical Hopf bifurcation. Figure 2C is a graph showing the change of period with the parameter *g*_*cantot*_. Within the considered range, the change of period is relatively complicated. Around *g_*cantot*_* = 0.45, the period jump sharply, which the reason for this is that there is also a period doubling bifurcation in Figure 2B.

**FIGURE 3:**
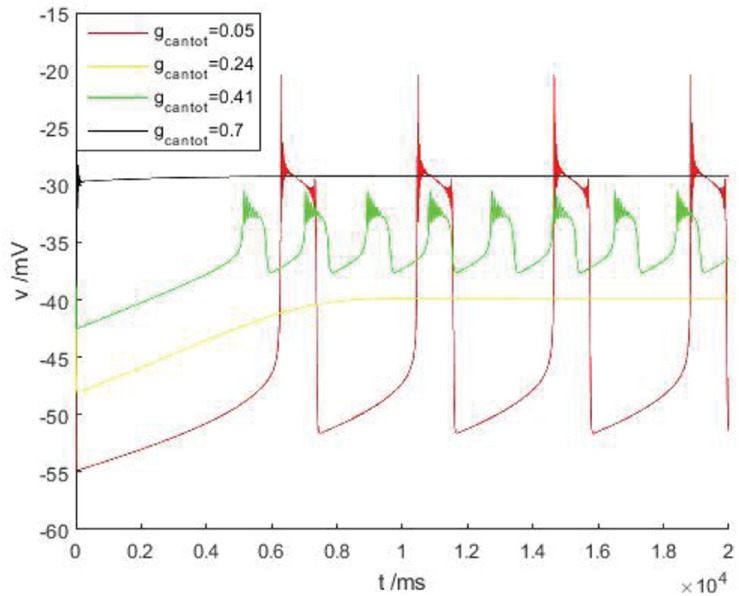
The time history figure of voltage of PBC neurons under different parameters without stimulus current. Red, orange, green, and black are the action potentials when the parameters *g*_*cantot*_ are 0.05, 0.24, 0.41, and 0.7, respectively.

Discussed below in Figure 2B the firing behavior of four ranges, respectively, take on four scope *g*_*cantot*_ for 0.05, 0.24, 0.41, and 0.7, as shown in Figure 3, it is known that cell is resting in II and IV area. Therefore, the firing pattern of parameters in regions I and III will be mainly discussed next.

**FIGURE 4:**
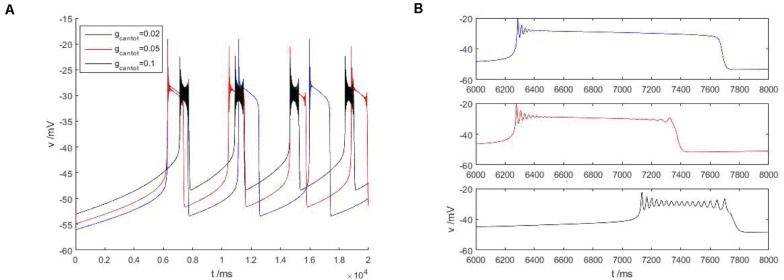
**(A)** Firing pattern of cells in region I of Figure 2B, *g*_*cantot*_ is 0.02, 0.05, and 0.1, respectively. **(B)** Is a partial enlarged view of periodic bursting corresponding to different parameters *g*_*cantot*_ in **(A)**.

As shown in Figure 4A, within the scope of the area I, with the increase of *g*_*cantot*_, the bursting phenomenon in the Figure 4 can be observed to transition from blue to red and then to black, and the spike number of buster also changes with the increase of *g*_*cantot*_. When *g*_*cantot*_ increases to the boundary of region I, namely *g*_*cantot*_ = 1, the firing of buster in the middle of the peak potential was gradually compressed, similar to a H-shape, the dynamics performance for near the point at which there is a small amplitude of limit cycle attractor, and is stable. In addition, with the increase of *g*_*cantot*_, the spike potential in each *g*_*cantot*_ also decreases. As can be seen from the local enlarged view of Figure 4B, *g*_*cantot*_ shows great differences in the resting state. When *g_*cantot*_* = 0.02, the action potential is disturbed at the initial stage of the resting state, and the amplitude gradually decreases until 0, and the number of spiking is 4. When *g_*cantot*_* = 0.05, the membrane potential is disturbed not only at the initial stage of resting state but also at the terminal stage, but the number of peak spike at this time is significantly higher than that of the former. When *g_*cantot*_* = 0.1, the membrane potential is disturbed in the whole resting phase, and the change frequency is non-monotonic. The number of peaks reaches 15, but the change amplitude of busters is smaller than that of the former two parameters. Different from the first, it can be seen in the latter two cases that the middle spike of the buster presents an H-shape.

**FIGURE 5:**
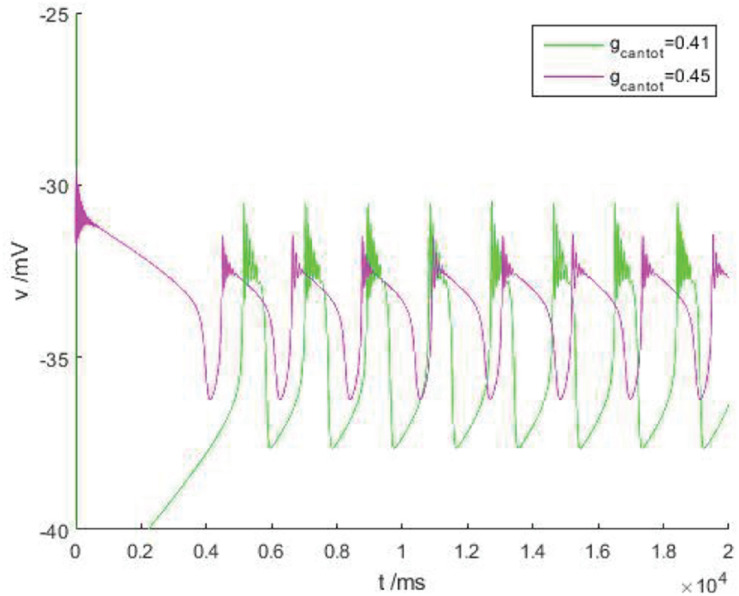
The burster of cells in area III, green for *g_*cantot*_* = 0.41, purple for *g*_*cantot*_ = 0.45.

In Figure 5, the green and purple are the action potentials of neuron when *g_*cantot*_* = 0.41 and 0.45, respectively. It can be observed that with the increase of *g*_*cantot*_ in region III, the spike potential gradually decreases while the frequency of bursting increases, which is completely consistent with the situation in Figure 2. In addition, it is obvious that different parameters will affect the number of peaks in the bursting, which can be found in Figure 4, and has been explained in detail. At this time, area III is basically similar, and its local diagram will not be introduced.

## Firing Analysis Based on Calcium Activated Conductance *g_cantot_* With Stimulated Current (*I_exc_* = −40)

The change of bursting in the absence of external stimulation current (*I_*exc*_* = 0) has been discussed previously for conductance *g*_*cantot*_. This section begins to discuss the influence of external stimulus current on the firing. *I_*exc*_* = -40 is taken here. It should be noted that the negative current here is not less than 0 in the mathematical sense, but the reference direction of the current is opposite.

**FIGURE 6:**
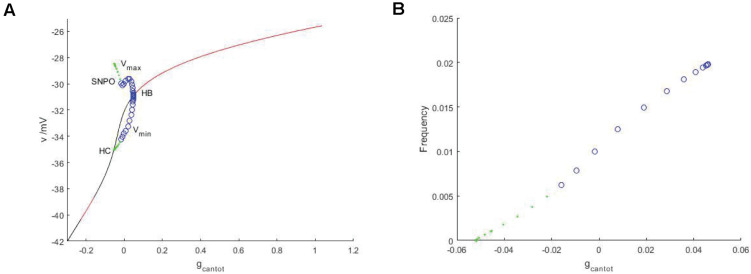
**(A)** Bifurcation diagram of potential at stimulus current *I_*exc*_* = –40 regarding parameter *g*_*cantot*_. The black and red represent the equilibrium points of instability and stability, respectively, the blue hollow circle represents the unstable periodic orbit, and the green solid circle represents the stable closed orbit. **(B)** Relationship between frequency and parameter *g*_*cantot*_. PBC based on stimulation current (*I_*exc*_* = –40).

Observe the bifurcation diagram of *g*_*cantot*_, as shown in Figure 6. Black and red represent the unstable and stable equilibrium points, respectively, while blue and green represent the unstable and stable limit cycles. Compared with Figure 2, the stability has undergone a great change. First, the transition mode of stability has a great difference, from the alternation of stability and instability to a single change. Therefore, Hopf bifurcation is generated at *g_*cantot*_* = 0.04611. The upper and lower branches of the periodic bifurcation curve correspond to the maximum and minimum values of the action potential, respectively, and lower branch of the curve extends to the equilibrium curve, which means that there is a homoclinic bifurcation. The period and frequency are reciprocal to each other. The essence of the problem is the same. The period of *g*_*cantot*_ is discussed in Figure 2, and frequency is discussed here. Figure 6B is frequencies, which is also very different from period in Figure 2C. After the stimulation current is applied, the frequency change monotonously, and the change rate always remains constant.

**FIGURE 7:**
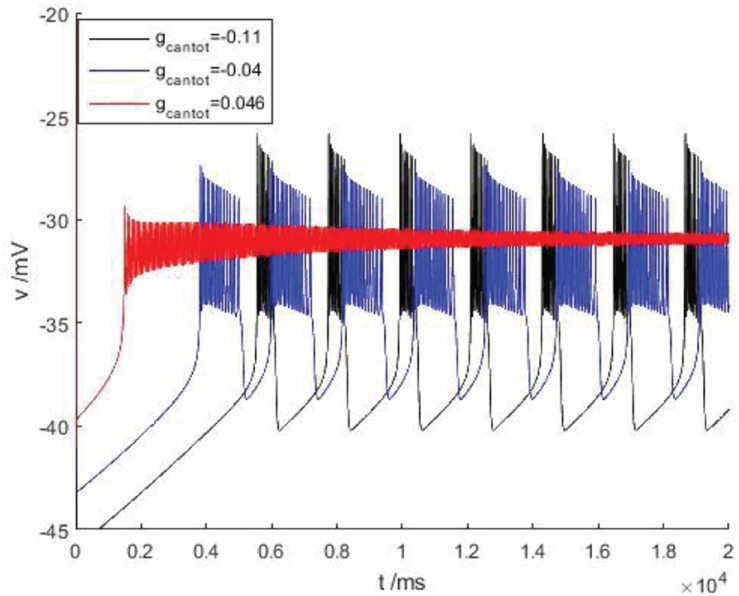
There is a stimulus current flowing down the cell at different parameters of bursting. Black, blue, and red are the membrane potentials when *g_*cantot*_* = –0.11, –0.04, and 0.046, respectively.

**FIGURE 8:**
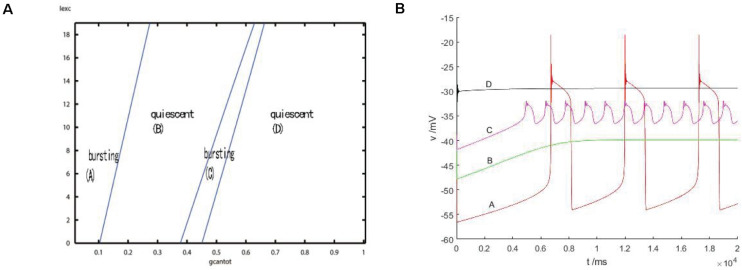
**(A)** activity patterns of neurons in a two-parameter space (*g*_*cantot*_,*I*_*exc*_). Space is divided into four parts: (A) burning, (B) quiet, (C) burning, and (D) quiet. **(B)** Firing in four parts: where (A) *g_*cantot*_* = 0.1,*I_*exc*_* = 10, (B) *g_*cantot*_* = 0.35, *I_*exc*_* = 10, (C) *g_*cantot*_* = 0.54,*I_*exc*_* = 10, and (D) *g_*cantot*_* = 0.8,*I_*exc*_* = 10.

After considering the external stimulus current, the stable state of its equilibrium points is obtained, and values are, respectively, taken from its unstable range to observe firing. As shown in Figure 7, with the increase of *g*_*cantot*_, membrane potential amplitude decreases, while the frequency gradually increases, and the number of peaks also increases. Finally, chaos phenomenon appears, which is quite different from that without considering the external stimulus current. With the increase of parameters, the membrane potential of the former gradually flattens out and even reaches a resting state, which is dynamically explained as a stable equilibrium point nearby, while the latter tends to oscillate intensively, corresponding to a stability limit cycle with a small amplitude in the dynamic system. Therefore, the former is called point bursting and the latter is called cycle bursting. Figure 8 discusses the effect of simultaneous changes of external stimulation current and conductance *g*_*cantot*_ on action potential. Its two-parameter diagram divides the region into four parts, of which (A,C) region is bursting and (B,D) is quiescent. This is consistent with the previously discussed case of no stimulation current. The former is a special case of this case.

## Fast and Slow Analysis

**FIGURE 9:**
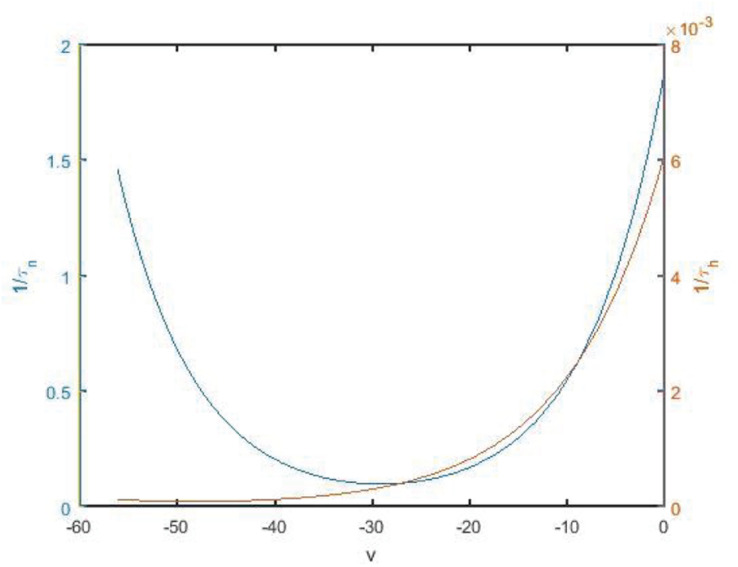
State function *1/τ_*n*_* and *1/τ_*h*_* diagram along with the change of membrane potential. Blue and red are the curves of *n* and *h*, corresponding to the left vertical axis and the right vertical axis, respectively.

The change rate of potassium ion channel is slower than that of sodium ion channel whether it is activated or deactivated. The spikes of the neuron are highly permeable to potassium ions, but hardly permeable to sodium ions, so that the membrane potential tends to potassium ions. As shown in Figure 9, 1/τn and 1/τ_*h*_ are time state functions of channel gates *n* and *h*, respectively. It can be found that the change of 1/τ_*h*_ is significantly slower than that of 1/τ_*n*_. Therefore, *h* is regarded as a slow parameter, and fast and slow dynamics analysis is carried out on fast subsystems (Teka et al., 2011, 2012).

By parameterizing the slow variable *h*, the original three-dimensional system becomes a two-dimensional fast subsystem only related to the fast variables *v* and *n*. By using time separation, the system can be analyzed using two-fast/one-slow technology. Make:

$$\text{S}=\left\{\left(v,h,n\right)\in R^{3}|f\,\left(v,h,n\right)=0\right\}$$

**FIGURE 10:**
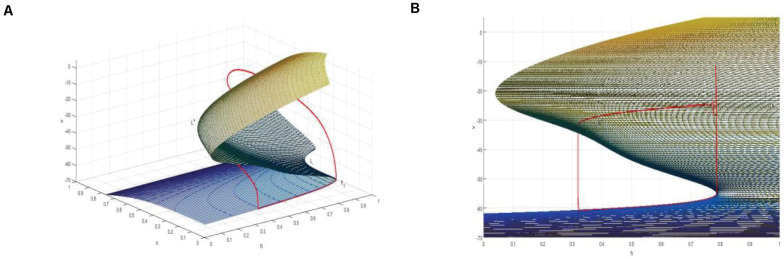
**(A)** The critical manifold and periodic orbit of full system for *I_*exc*_* = 16, *g_*cantot*_* = 0.02. *L*^+^ and *L*^–^ are the upper and lower fold curves, respectively. The red is the periodic orbit of the full system superimposed on the critical manifold, where, *F*_1_ is the bifurcation point on the fold curve. **(B)** The projection of the critical manifold on the (*v*,*n*) plane along the *n*-axis direction.

Thus, a two-dimensional critical manifold is obtained in a three-dimensional space. As shown in Figure 10A, the critical manifold is divided into three parts by a fold curve (*L*^+^, *L*^–^), the lower sheet and the upper sheet are attracting, the middle sheet is repulsed, and the folding curve satisfies

$$L^{\pm }=\left\{\left(v,h,n\right)\in R^{3}|f\,\left(v,h,n\right)=0,\frac{\partial f}{\partial v}\,\left(v,h,n\right)=0\right\}$$

**FIGURE 11:**
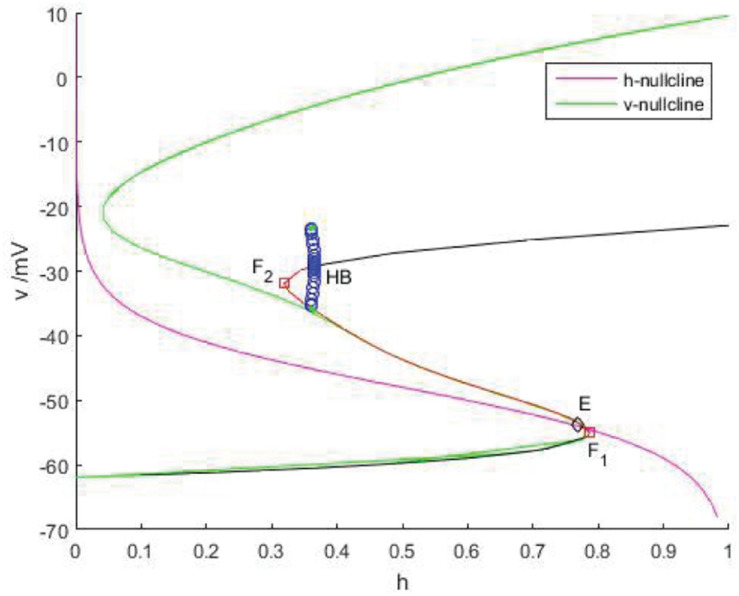
The phase plane of the fast subsystem with *g*_*cantot*_ = 0.02, *I*_*exc*_ = 16. *v*-nullcline (green) and *h*-nullcline (red) intersect at point E (diamond). Another S-curve is the bifurcation curve of the equilibrium point. On this curve, black indicates the stable equilibrium, red represents the unstable equilibrium, *F*_1_ and *F*_2_ (squares) are fold and HB is Hopf bifurcation.

As shown in Figure 11, *v*-nullcline is S-shaped and consists of three branches. The lower branch, middle branch and upper branch correspond to resting, oscillating and tonic states. The intersection point of *h*-nullcline and *v*-nullcline is the equilibrium point of the fast subsystem. When the equilibrium point is located in the middle branch, the cell oscillated, which is the premise of producing bursting. The bifurcation curve of the equilibrium point also has three branches. The lower branch consists of the stable focus of hyperpolarized state, and the middle branch consists of unstable node. The system is bistable between the lower and upper branch for a range of *h* values. In the upper branch, the equilibrium point changes from unstable to stable with the increase of *h*, resulting in Hopf bifurcation. The periodic orbit bifurcation curve from this point terminates at the middle branch, resulting in homoclinic bifurcation. Through comparison, it can be seen that the bifurcation curve of the equilibrium point is almost consistent with the trend of *v*-nullcline. First, *v*-nullcline is obtained from $\frac{d\,v}{d\,t}=0$, namely satisfies *f*(*v*,*h*,*n*) = 0. The equilibrium point curve of the fast subsystem also requires $\frac{d\,v}{d\,t}=0$, therefore, the lower branch and the middle branch of the fast subsystem almost coincide with each other, but for the bifurcation curve of the equilibrium point of a two-dimensional system, not only one equation is required to be zero, but also the other equation is required to be zero, namely $\frac{d\,n}{d\,t}=0$, which is the requirement that *v*-nullcline does not need to meet, so the two systems show great deviation in the upper branch. If the two systems are required to be progressive in the upper branch, they are equivalent to (*n*_∞_(*v*)−*n*)/τ_*n*_(*v*)≡0, and then there is 1/τ_*n*_(*v*)→0, in other words,τ_*n*_(*v*)→∞. It can be seen from Figure 9 that 1/τ_*n*_(*v*) gradually increases and can no longer approach zero after reaching the depolarized state.

Therefore, *v*-nullcline and the upper branch of the equilibrium point curve show great difference. In fact, *v*-nullcline is also the curve where the singular manifold intersects with an interface in Figure 10A. The singular manifold has an S-shape, and *v*-nullcline naturally has an S-shape. In Figure 10B, the periodic orbit moves along the lower branch of the invariant manifold, jumps up through the bifurcation point *F*_1_ on the curve *L*^+^, and oscillates in the middle branch again through the bifurcation point to the upper branch, and its direction is similar to the bifurcation line of the equilibrium point. Next, the bursting of the system is analyzed in the phase plane (*h*,*v*).

### Fold/fold Bursting

**FIGURE 12:**
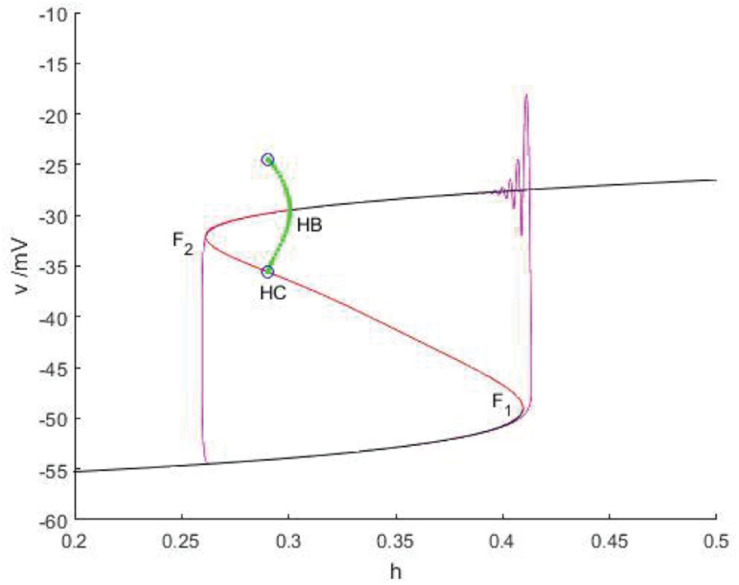
When *I_*exc*_* = 12, *g_*cantot*_* = 0.11, the constitutional diagram of phase diagram of fast subsystem and bifurcation diagram of slow variable. Black and red are the stable and unstable equilibrium points, respectively, green and blue represent the stable and unstable limit cycles, and purple is the phase trajectory. F_1_ and F_2_ is the fold bifurcation, HB is the Hopf bifurcation, and HC is the homoclinic bifurcation.

When *I_*exc*_* = 12 and *g_*cantot*_* = 0.11, as shown in Figure 12, the equilibrium point curve is an S-shaped curve, bounded by the bifurcation point. The lower branch is composed of stable nodes, the middle branch is composed of unstable saddle points, the upper branch is composed of unstable saddle points passing through the Hopf bifurcation, from which a stable limit cycle is generated, and turning into stable focus. With the increase of the slow parameter *h*, the resting state of the lower branch corresponding to the equilibrium curve is shifted upward to the release state corresponding to the vicinity of the limit cycle through the fold bifurcation *F*_1_. The upper branch oscillates and weakens with the decrease of *h*, finally returns to the resting state through *F*_2_, and the firing ends. In this case, Hopf bifurcation has no influence on bursting. Therefore, this firing type is fold/fold bursting.

**FIGURE 13:**
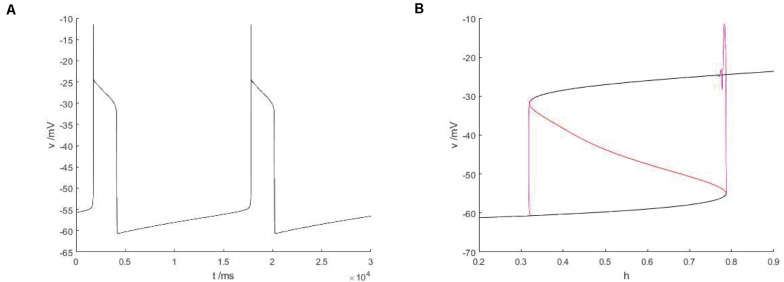
The constitutional diagram of the bifurcation parameters, where slow variable H are taken as bifurcation parameters, between the phase trajectory corresponding to the membrane potential of the fast subsystem. **(A)** Time history of membrane potential when *I_*exc*_* = 16 and *g_*cantot*_* = 0.02. **(B)** The bifurcation diagram of action potential with respect to *h* and the constitutional diagram of corresponding phase trajectories in diagram **(A)** show that black is a stable equilibrium points, red is an unstable equilibrium points, green is a stable limit cycle, blue is an unstable limit cycle, and purple is a phase trajectory.

As shown in Figure 13B, the equilibrium curve is still S-shaped and consists of three parts. The three branches are, respectively, composed of stable nodes, unstable equilibrium points and stable focal points. The phase diagram corresponding to bursting in Figure 13A is superimposed on the equilibrium curve to obtain the slow parameter analysis diagram of the fast subsystem. Under this parameter condition, there is no Hopf bifurcation in the bifurcation curve of the fast subsystem, so there is no stable limit cycle corresponding to the firing state. Therefore, it is not necessary to discuss the bifurcation type of transition between the quiescent state and the firing state, but only the bifurcation related to the hysteresis loop. From the Figure 13B, it can be found that the lower state shifts to the upper state via the fold bifurcation with the increase of bifurcation parameters, and then the upper state returns to the lower state via another fold bifurcation point until the firing ends. At this time, the firing type of the system is fold/fold bursting. Compared with Figure 12, both cases are the same type of firing, but for this type of bursting, Hopf bifurcation plays no key role and will not affect the type of bursting. In the former, there is Hopf bifurcation, while in the latter, Hopf bifurcation gradually approaches the fold bifurcation until it disappears. Fold bifurcation are points on the fold curve, thus satisfying $\frac{d\,v}{d\,t}=0$ and $\frac{\partial f}{\partial v}=0$, while Jacobian matrix of fast subsystem is $J=\left(\begin{array}{cc} \frac{\partial f}{\partial v} & \frac{\partial f}{\partial n}\\ \frac{\partial g}{\partial v} & \frac{\partial g}{\partial n} \end{array}\right)$.

Therefore, it is only necessary to satisfy $t\,r\,a\,c\,e\,\left(J\right)=\frac{\partial f}{\partial v}+\frac{\partial g}{\partial n}=0$ at the Hopf bifurcation, which already exists, so only $\frac{\partial f}{\partial v}=0$ is needed, and when $\frac{\partial g}{\partial n}=0$, that is $\frac{1}{\tau_{n}\,\left(v\right)}\to 0$, there is a Hopf bifurcation coinciding with the fold bifurcation.

### Fold/homoclinic Bursting

**FIGURE 14:**
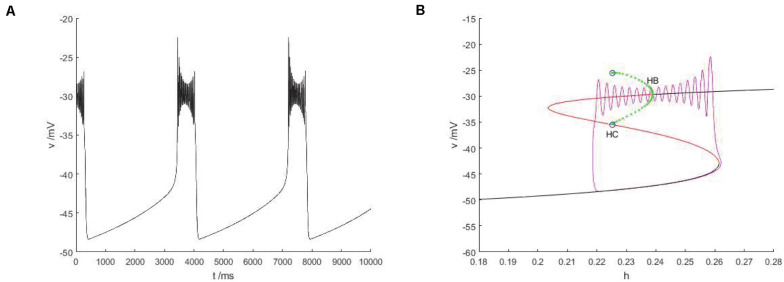
The constitutional diagram of bifurcation parameters of phase trajectory corresponding to membrane potential of fast subsystem and slow variable *h*. **(A)** the time history diagram of membrane potential when *I_*exc*_* = 0 and *g_*cantot*_* = 0.1. **(B)** The bifurcation diagram of action potential with respect to *h* and the combination diagram of corresponding phase trajectories in diagram **(A)** show that black is a stable equilibrium points, red is an unstable equilibrium points, green is a stable limit cycle, blue is an unstable limit cycle, and purple is a phase trajectory.

The slow variable is regarded as a bifurcation parameter, and the fast subsystem is analyzed to obtain Figure 14B. As shown in the figure, the equilibrium point curve of the fast subsystem is composed of three parts, namely S-shape, the lower branch (black) is stable node, the middle branch is unstable saddle point, the upper branch are composed of unstable saddles point and stable nodes, and the Hopf bifurcation is taken as a boundary, from which a limit cycle of stable points appears. At the same time, the phase diagram corresponding to the membrane potential in Figure 14A is superimposed on the equilibrium point curve of the fast subsystem to analyze the firing type under this parameter.

With the increase of the control variable h, the stable node corresponding to the resting state of the lower branch of the equilibrium bifurcation curve disappears, and is transformed into an unstable saddle point through the folding bifurcation, and then transferred upward to the vicinity of the stable limit cycle corresponding to the firing state. The stable limit cycle starting from the Hopf bifurcation point gradually approaches the saddle point branch of the equilibrium curve with the decrease of the slow parameter h, and returns to the stable node corresponding to the resting state near the saddle point homoclinic orbit, and the firing activity ends. Therefore, the firing type in which the rest state and the firing state change with each other is fold/homoclinic bursting.

### Hopf/homoclinic Bursting

**FIGURE 15:**
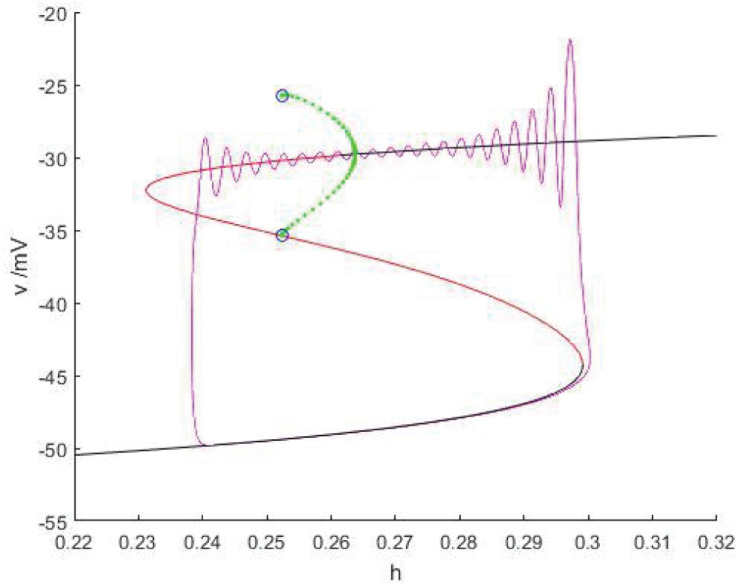
When *I_*exc*_* = 17 and *g_*cantot*_* = 0.2416, the phase trajectory corresponding to the membrane potential of the fast subsystem and the slow variable *h* are superimposed as bifurcation parameters.

As shown in Figure 15, when *I_*exc*_* = 17 and *g_*cantot*_* = 0.2416, the equilibrium point curve is S-shaped, the lower branch corresponds to the resting state, jumps to the upper branch through the fold bifurcation *F*_1_, the motion trajectory generates damped oscillation near the stable focus, then passes through the Hopf bifurcation (*h* = 0.2637), and the final firing state is transferred to the resting state through the homoclinic orbit bifurcation of the limit cycle with the decrease of the slow variable *h*, and the final firing is finished. Therefore, this kind of firing mode is Hopf/homoclinic bursting.

## Conclusion

In this study, a single-compartment PBC neuron model is obtained by simplifying TB model, and Based on this model, the effects of different calcium-activated non-specific cation current (*I*_*CaN*_) and external stimulus current (*I*_*exc*_) on membrane potential are investigated. Firstly, by adjusting the calcium activated conductance (*g*_*cantot*_), it is found that the number of peaks in bursting pattern changes with the parameters. Then the stimulation current was adjusted to observe the discharge difference caused by calcium activated conductance. The plane is divided into four regions in parameter space (*g*_*cantot*_, *I*_*exc*_), and different regions were corresponding to the resting state and active state, respectively, which provided a more comprehensive understanding of the parameter region of neuronal firing. Finally, by using the fast-slow analysis, the slow variables were parameterized and the parameters *g*_*cantot*_ and *I*_*exc*_ were changed, respectively, to discuss the dynamic mechanism of the firing pattern of PBC neuron, and three types of bursting was found, which are fold/fold bursting, fold/homoclinic bursting and Hopf/homoclinic bursting, respectively.

This study shows that the external stimulation current has an important influence on the firing rhythm of PBC neuron, and the neuron can produce bursting by changing the calcium activation conductance. In the respiratory rhythm of mammals, there are many kinds of bursting patterns (Butera et al., 1999; Wang and Rubin, 2016). Through the experimental results and numerical simulation, it is also observed that there are abundant bifurcation phenomena in the model, such as Hopf bifurcation, folding bifurcation, homoclinic orbital bifurcation, saddle node bifurcation on the invariant ring and period-doubling bifurcation. Therefore, the research in this article is a great significance for understanding the dynamic mechanisms of PBC neural network, and it also provides some meaningful thoughts and opinions for further to explore the mechanisms of respiratory rhythms.

## Data Availability Statement

The raw data supporting the conclusions of this article will be made available by the authors, without undue reservation.

## Author Contributions

QY and JX designed and coordinated the study, and drafted the manuscript. QY, JX, and HC carried out the numerical simulation. QY and HC edited the manuscript. All authors gave final approval for publication.

## Conflict of Interest

The authors declare that the research was conducted in the absence of any commercial or financial relationships that could be construed as a potential conflict of interest.
